# Supplementary figures and images for: Tibetan PHD2D4E;C127S variant protects from viral diseases in hypoxia, but predispose to infections in normoxia via HIFα:IFN axis
Source: PLoS Pathog. 2025 Jun 26;21(6):e1013296. doi: 10.1371/journal.ppat.1013296 (PMC12212868; doi:10.1371/journal.ppat.1013296)

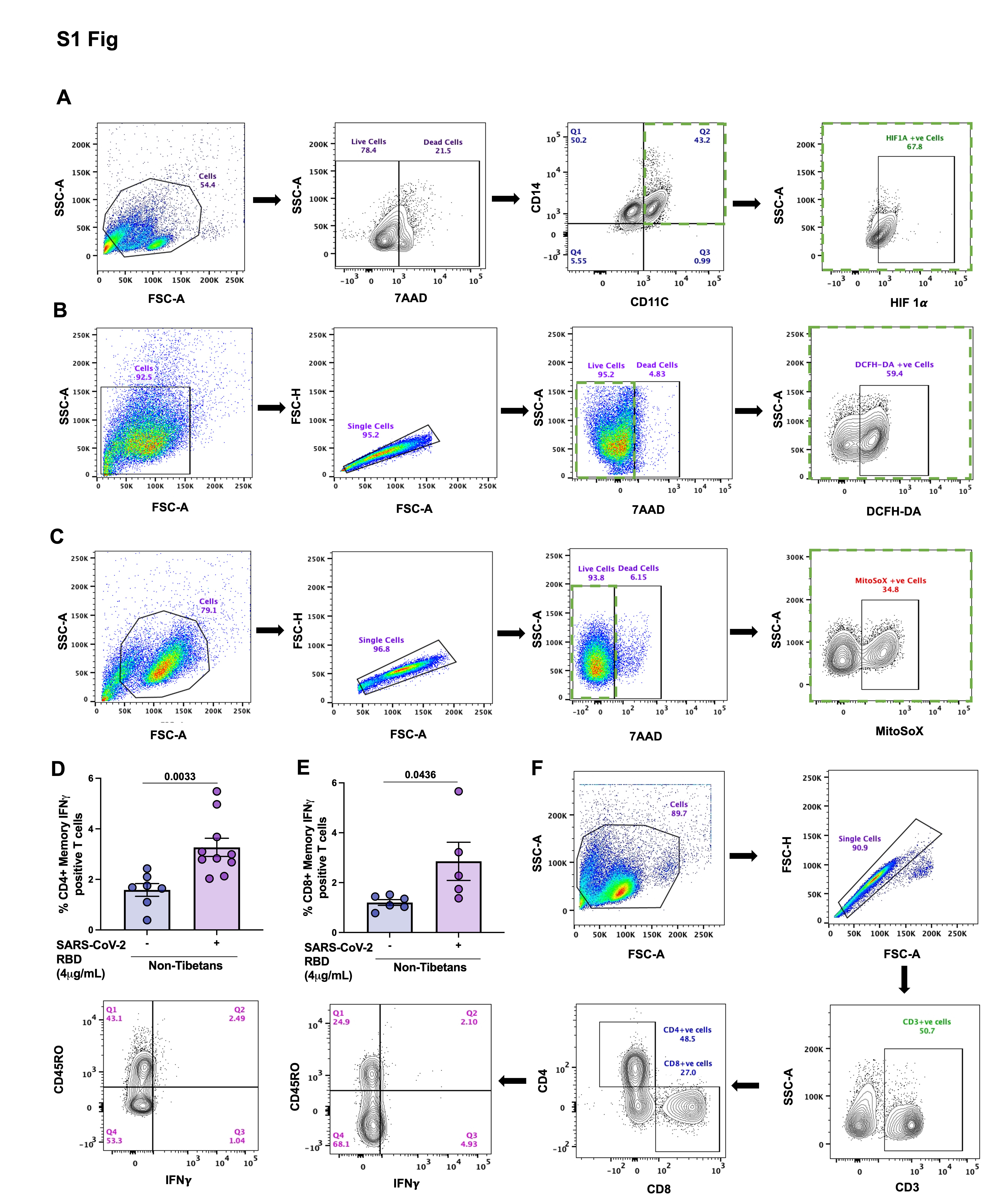

Supplement: S1 Fig — A. Gating strategy for HIF1a positive primary monocytes. DENV2 infected PBMCs from PHD2WT and PHD2D4E;C127S individuals, were stained with CD14-PE and CD11c-V450, and intracellular HIF1a (using primary and goat anti-rabbit AF488 secondary antibodies). B-C. Gating strategy for DCFH-DA (total ROS measurement) and MitoSOX (mitochondrial ROS measurement) positive cells. D. IFNγ positive CD4+ and, E. CD8+ Memory T cells in Non- Tibetan individuals were measured. Unpaired t test was used for analysis. Each dot represents a single individual. Data are mean ± SEM, P values were mentioned. F. Gating strategy for IFNγ+ T cells. PBMCs from PHD2WT and PHD2D4E;C127S individuals were stained for CD3 APC, CD4 V450, CD8 PerCP, CD45RO PE-Cy-7 and intracellular IFNγ PE markers. (TIFF) [file ppat.1013296.s001.tiff]

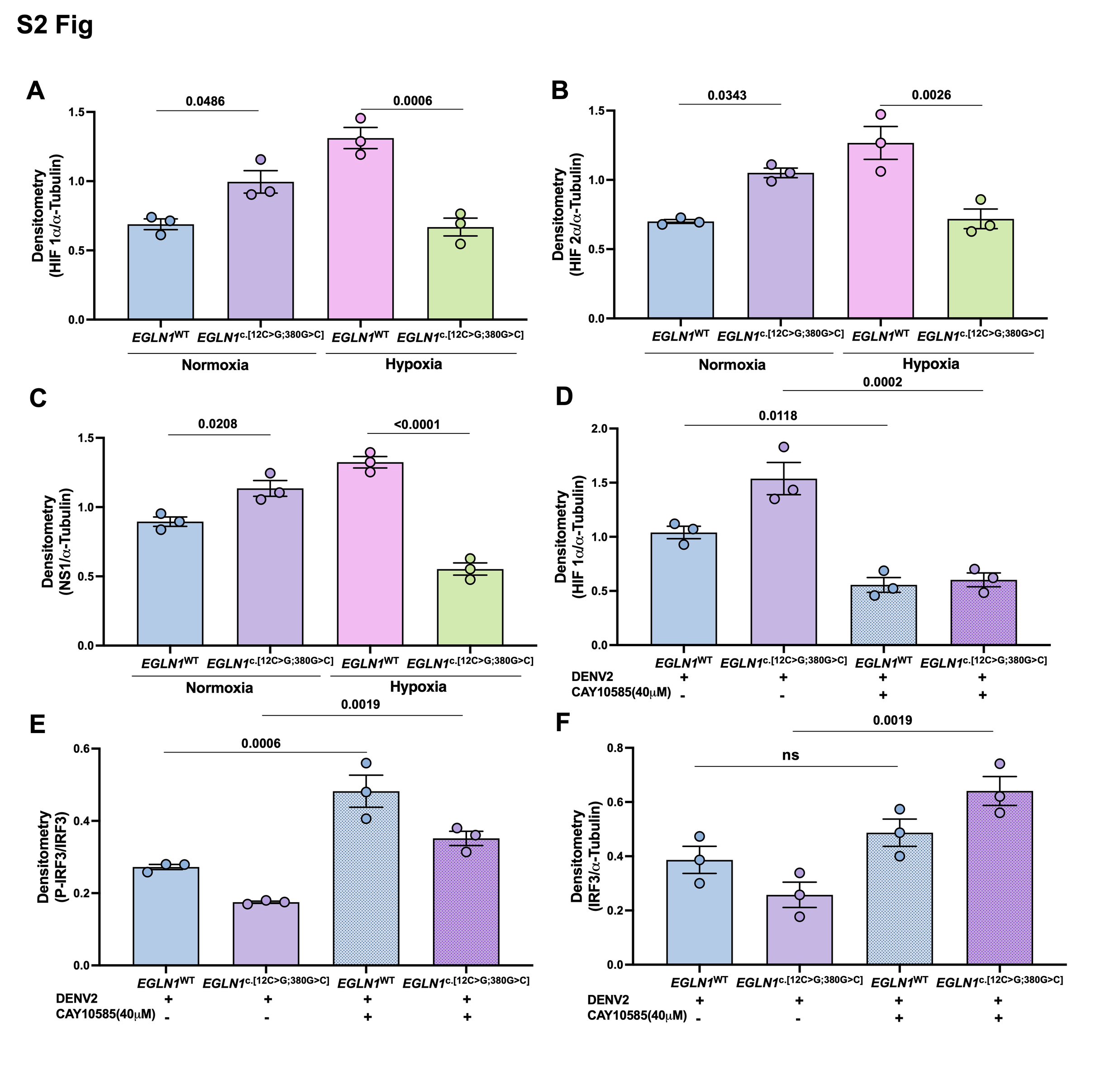

Supplement: S2 Fig — Densitometry analysis of the protein bands were analysed using the Image-J software; main Fig 2M (S2A – S2C Fig; one-way ANOVA followed by Bonferroni’s post-test was used), Fig 4B (S2D – S2F Fig; one-way ANOVA followed by Bonferroni’s post-test was used), Data are mean ± SEM from triplicate blots. P-values are mentioned in the graphs. (TIF) [file ppat.1013296.s002.tif]

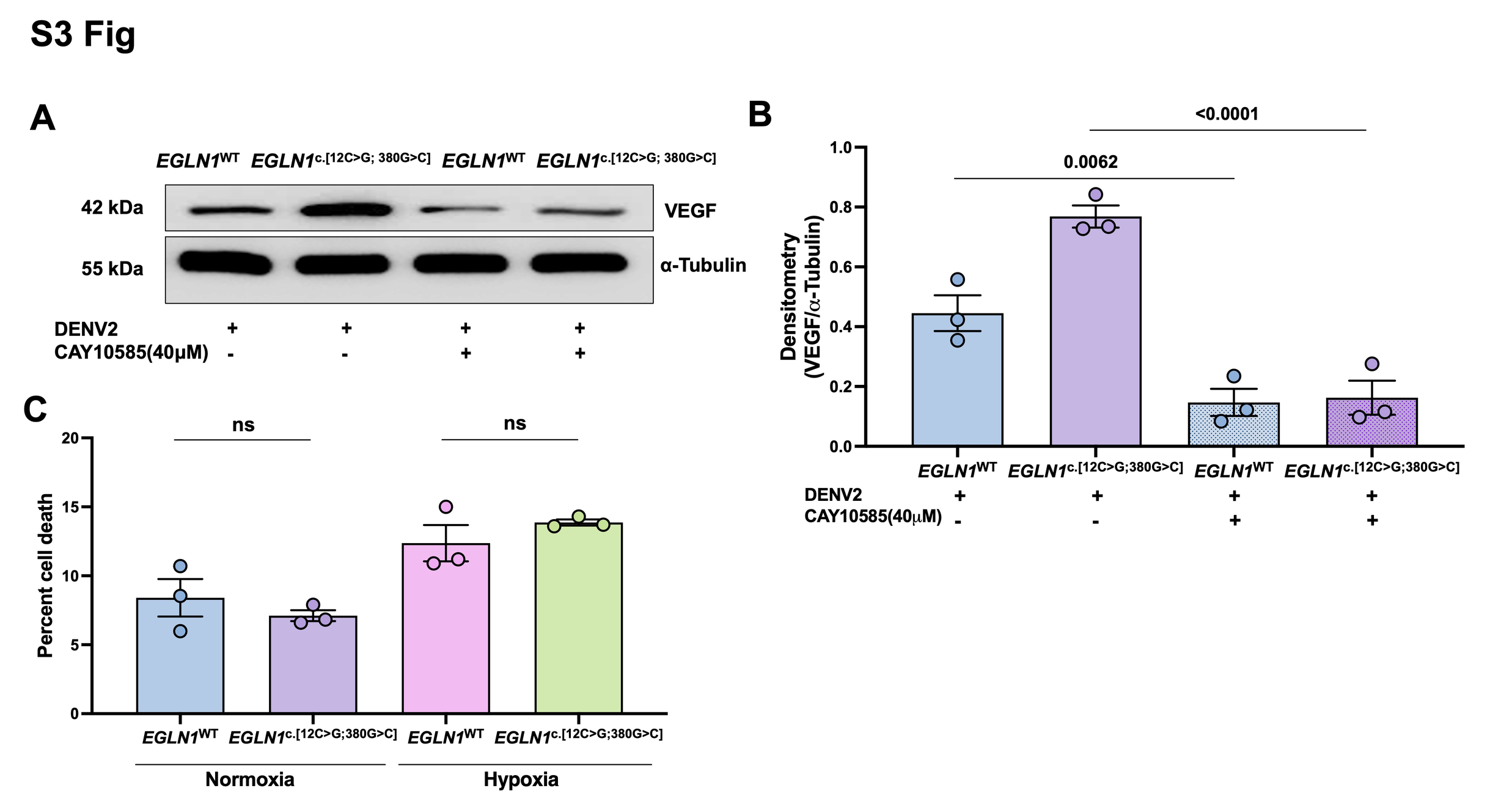

Supplement: S3 Fig — Expression of HIF target molecule, A.VEGF was measured through western blot in EGLN1c.[12C>G; 380G>C] or EGLN1WT expressing U937 cells infected with DENV2 as mentioned in Fig 4, in presence of HIFα inhibitor (CAY10585, 40μM). B. Densitometry analysis from 3 independent experiments of the protein band of VEGF, normalized with α-Tubulin. Data are mean ± SEM, one-way ANOVA followed by Bonferroni’s post-test was used. C. Cell death assay using Propidium iodide (100μg/μL) for staining EGLN1c.[12C>G; 380G>C] or EGLN1WT expressing U937 cells infected with DENV2 under normoxia and hypoxic conditions. Data are mean ± SEM from 3 independent experiments. One-way ANOVA and Bonferroni’s post-test were used for analysis. P-values are mentioned in the graphs. (TIF) [file ppat.1013296.s003.tif]

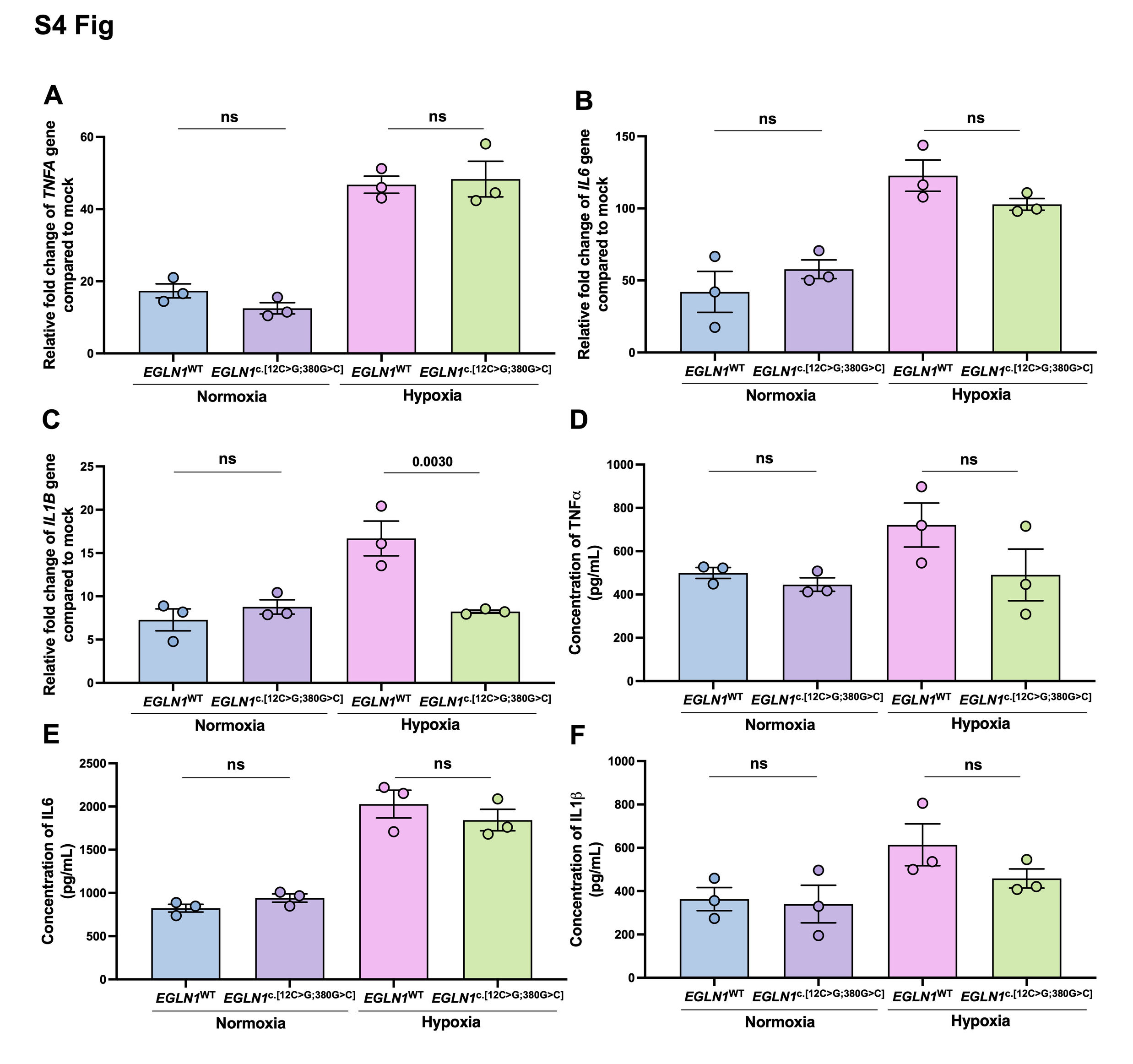

Supplement: S4 Fig — A-C. Pro-inflammatory cytokines TNFA, IL6 and IL1B were measured using qRT-PCR from EGLN1WT(PHD2WT) or EGLN1c.[12C>G; 380G>C] (PHD2D4E;C127S) experimental cell pellet (infected with DENV2) mentioned in Fig 2. Relative fold-change after normalization with human α-Tubulin. Data are mean ± SEM, One-way ANOVA and Bonferroni’s post-test were used for analysis. D–F. Proinflammatory cytokines TNFα, IL6 and IL1β protein levels were measured using CBA from above cell supernatants. Data are mean ± SEM, one-way ANOVA and Bonferroni’s post-test were used. P values are mentioned in the graphs. (TIF) [file ppat.1013296.s004.tif]

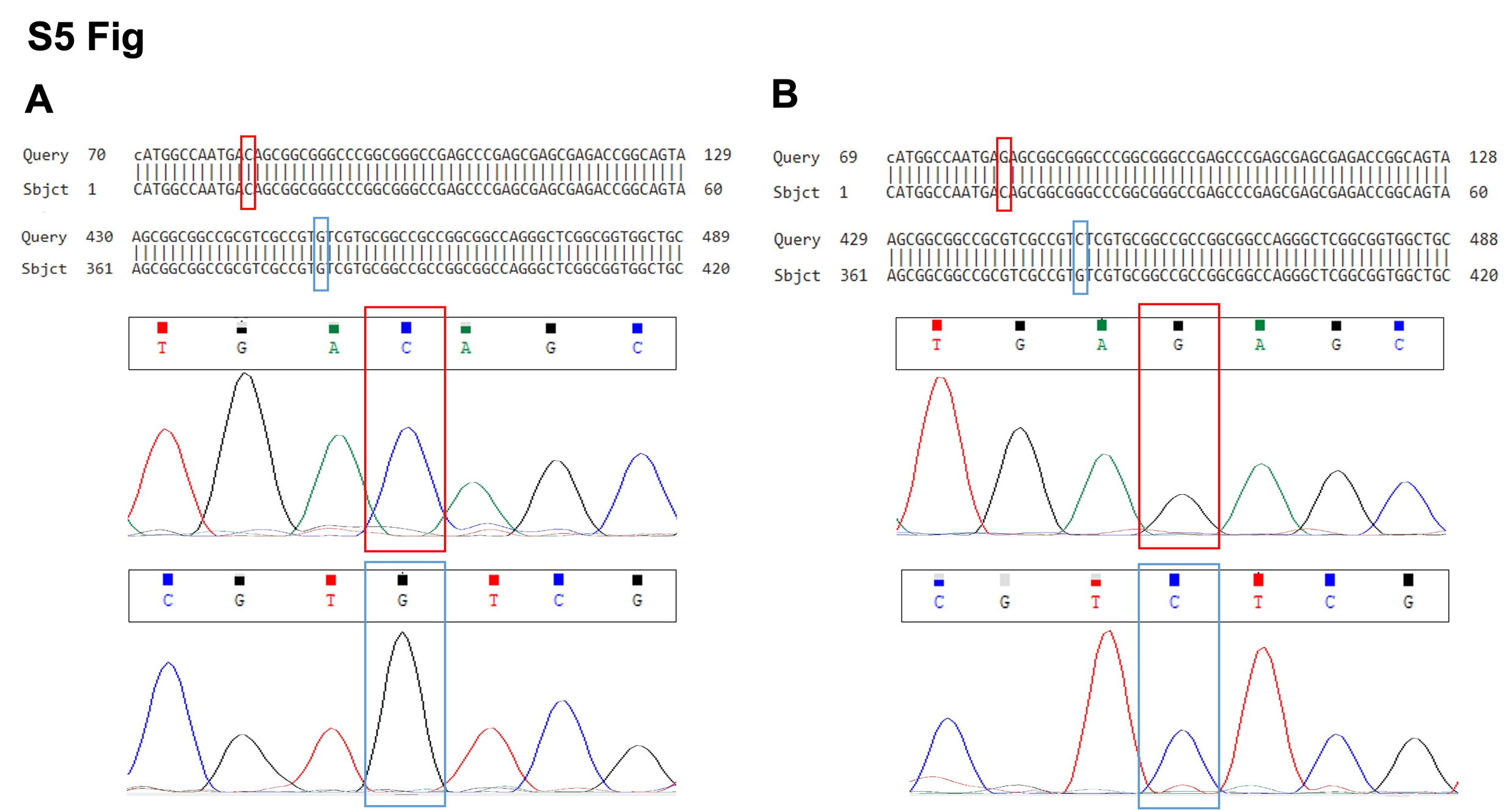

Supplement: S5 Fig — [12C>G; 380G>C] (PHD2D4E;C127S) in U937 cells. Sequencing/Genotyping of EGLN1WT or EGLN1c.[12C>G; 380G>C] in A-B. U937 cells. (TIF) [file ppat.1013296.s005.tif]

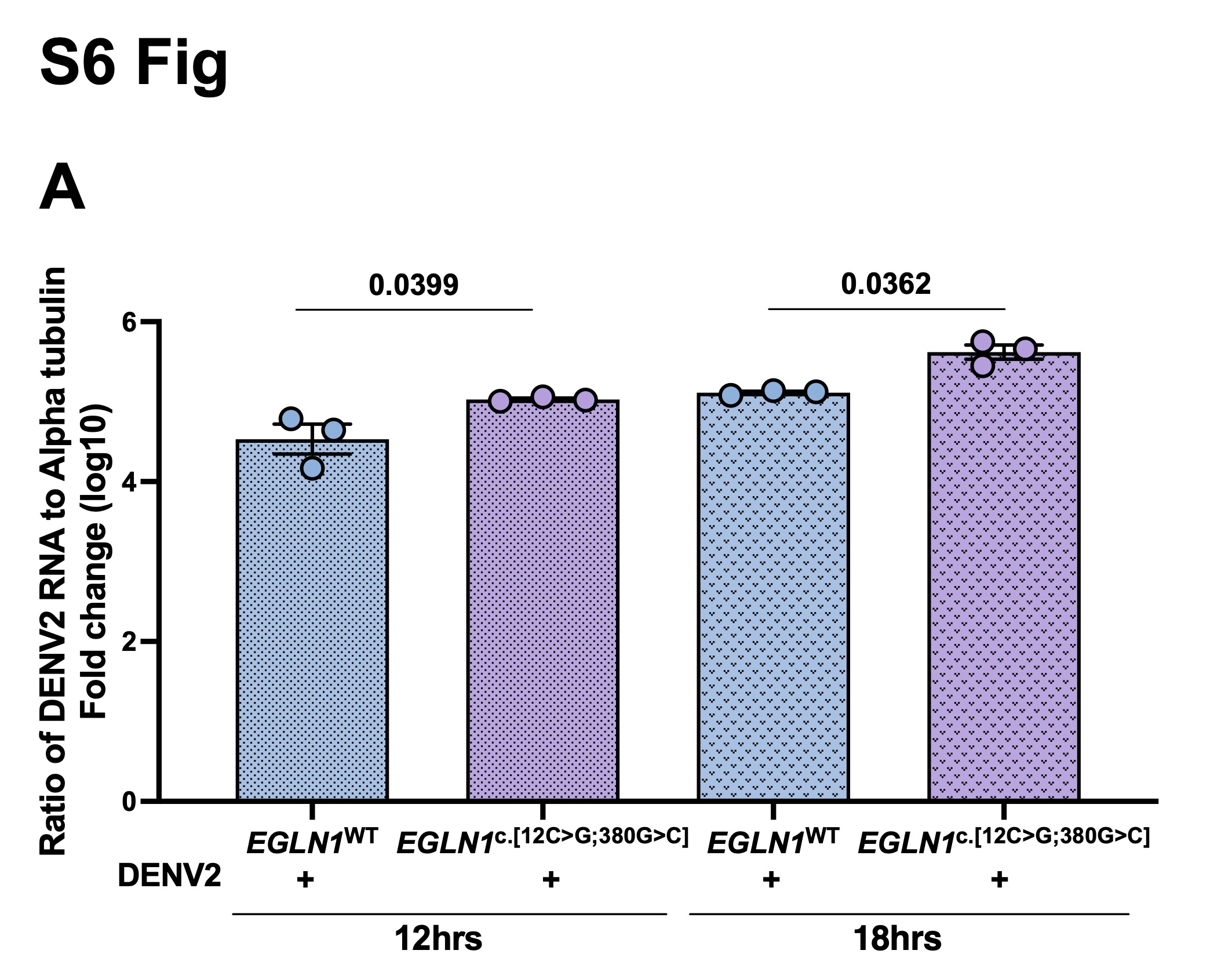

Supplement: S6 Fig — U937 monocytic cell lines expressing EGLN1c.[12C>G; 380G>C] or EGLN1WT were infected with DENV2 (MOI ~ 3) and incubated for 12hrs and 18hrs under normoxia (21% O2). Cells were collected and A. DENV2 RNA levels were measured using qRT-PCR, Data are mean ± SEM from 3 independent experiments, one-way ANOVA and Bonferroni’s post-test were used for statistical analysis. (TIF) [file ppat.1013296.s006.tif]

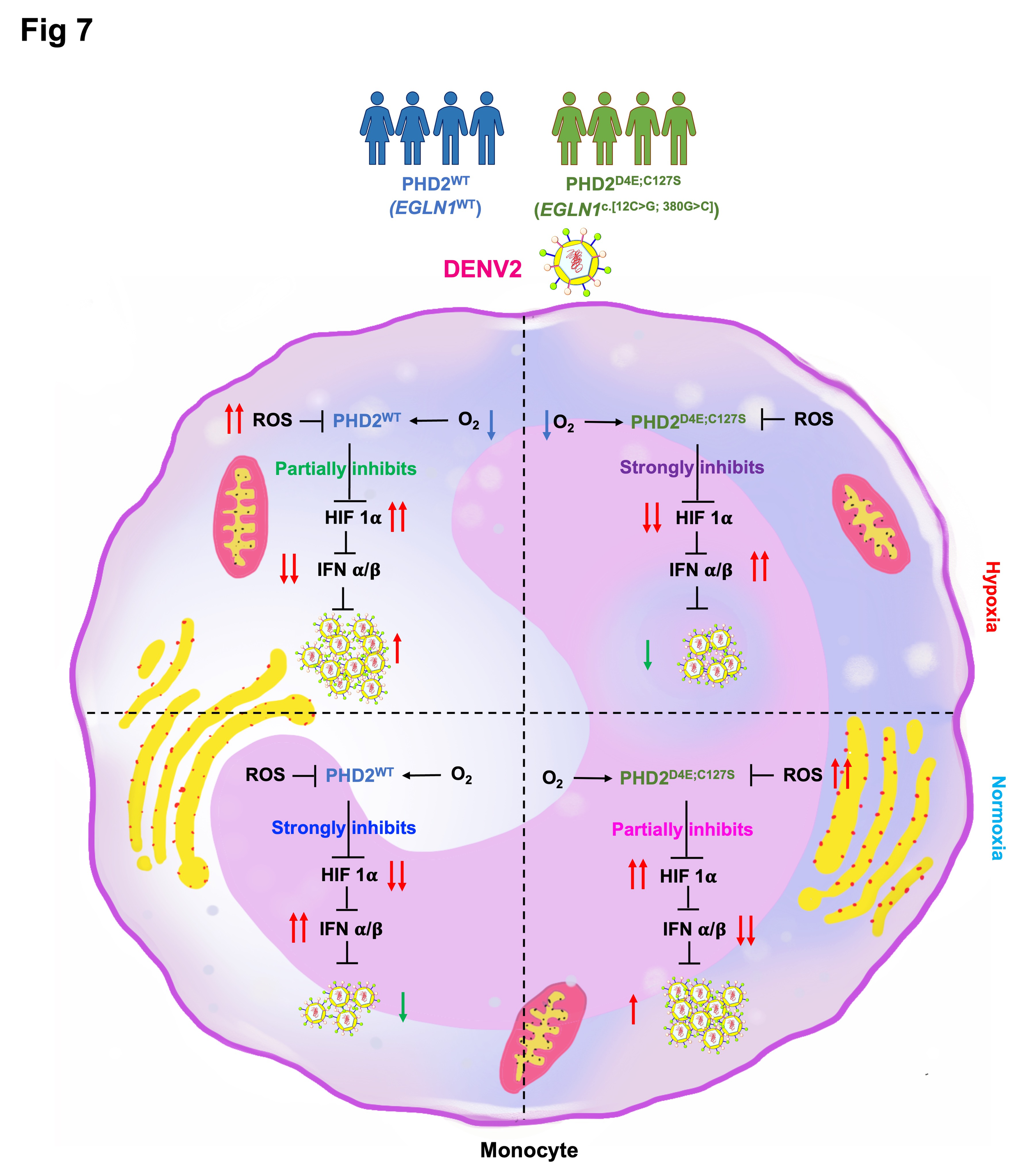

Supplement: S7 Fig — (TIF) [file ppat.1013296.s007.tif]

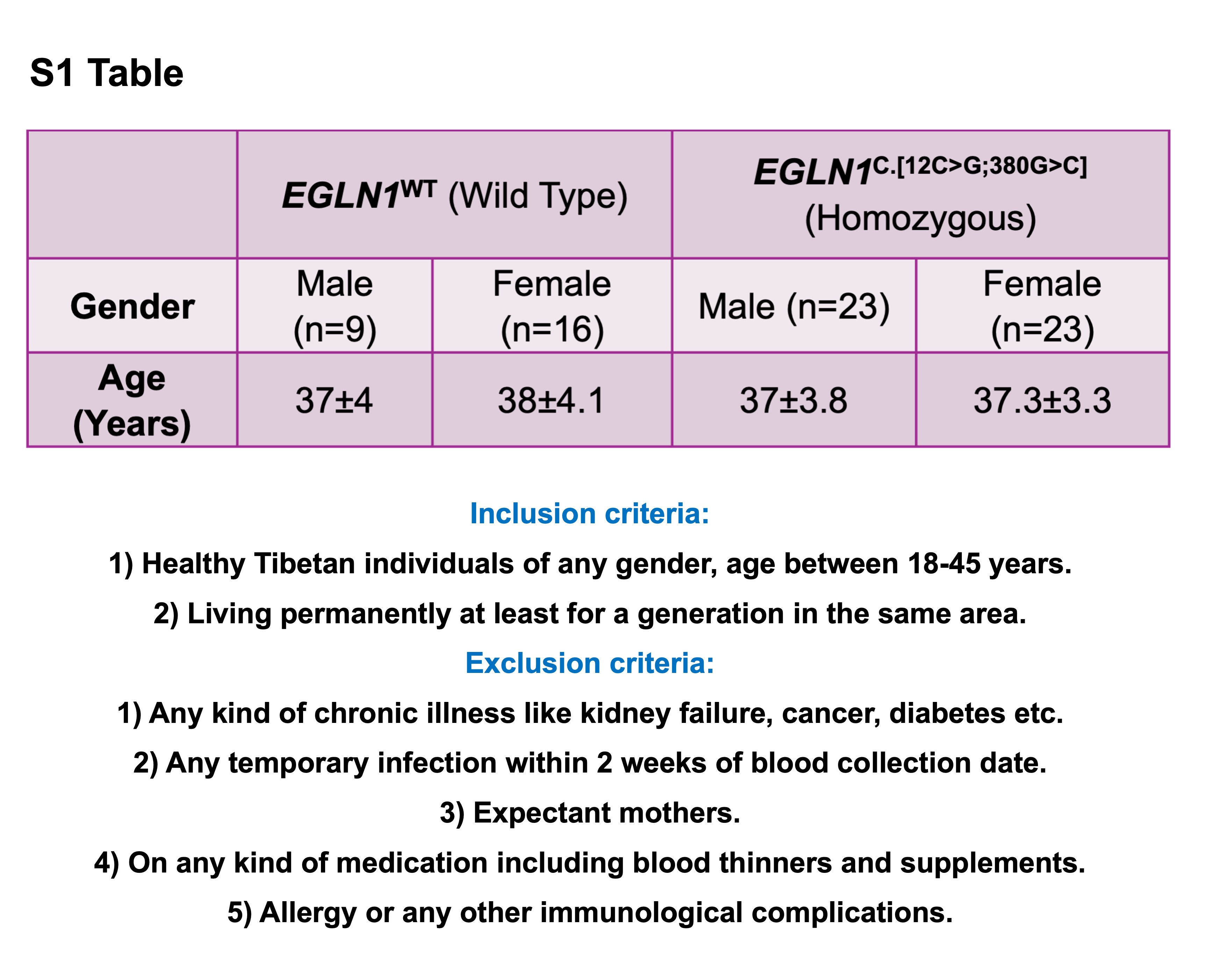

Supplement: S1 Table — (TIFF) [file ppat.1013296.s008.tiff]

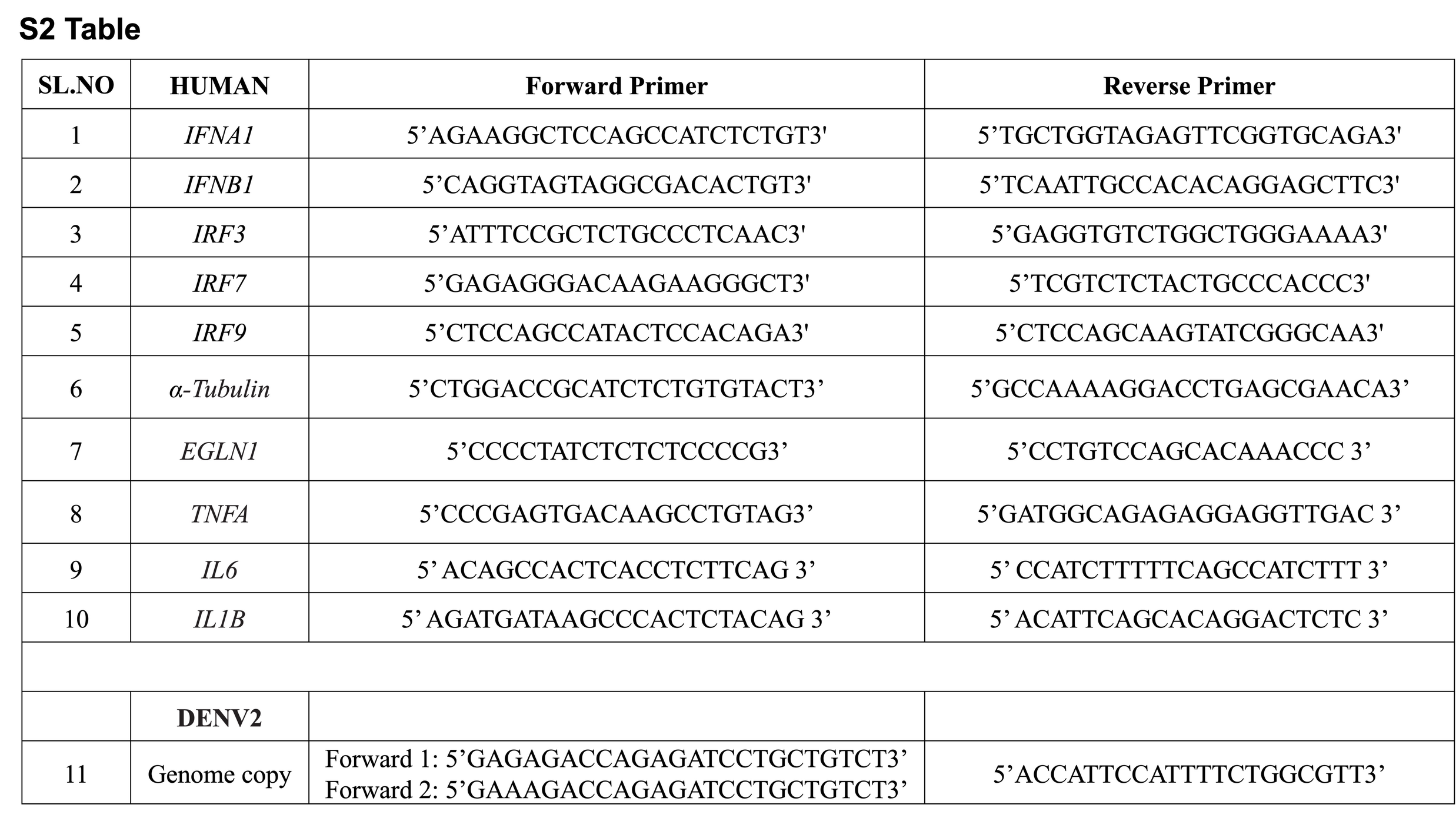

Supplement: S2 Table — (TIF) [file ppat.1013296.s009.tif]

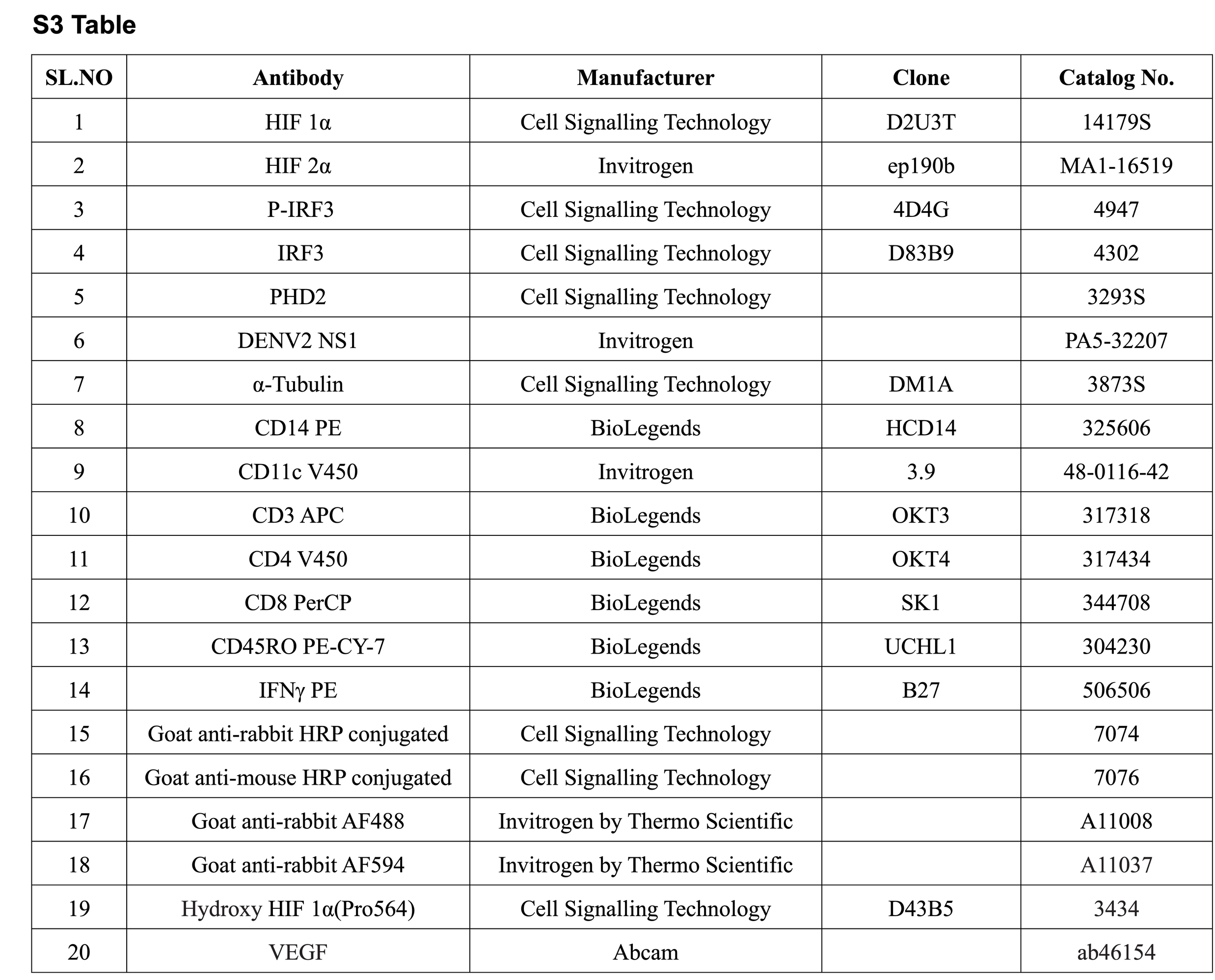

Supplement: S3 Table — (TIF) [file ppat.1013296.s010.tif]

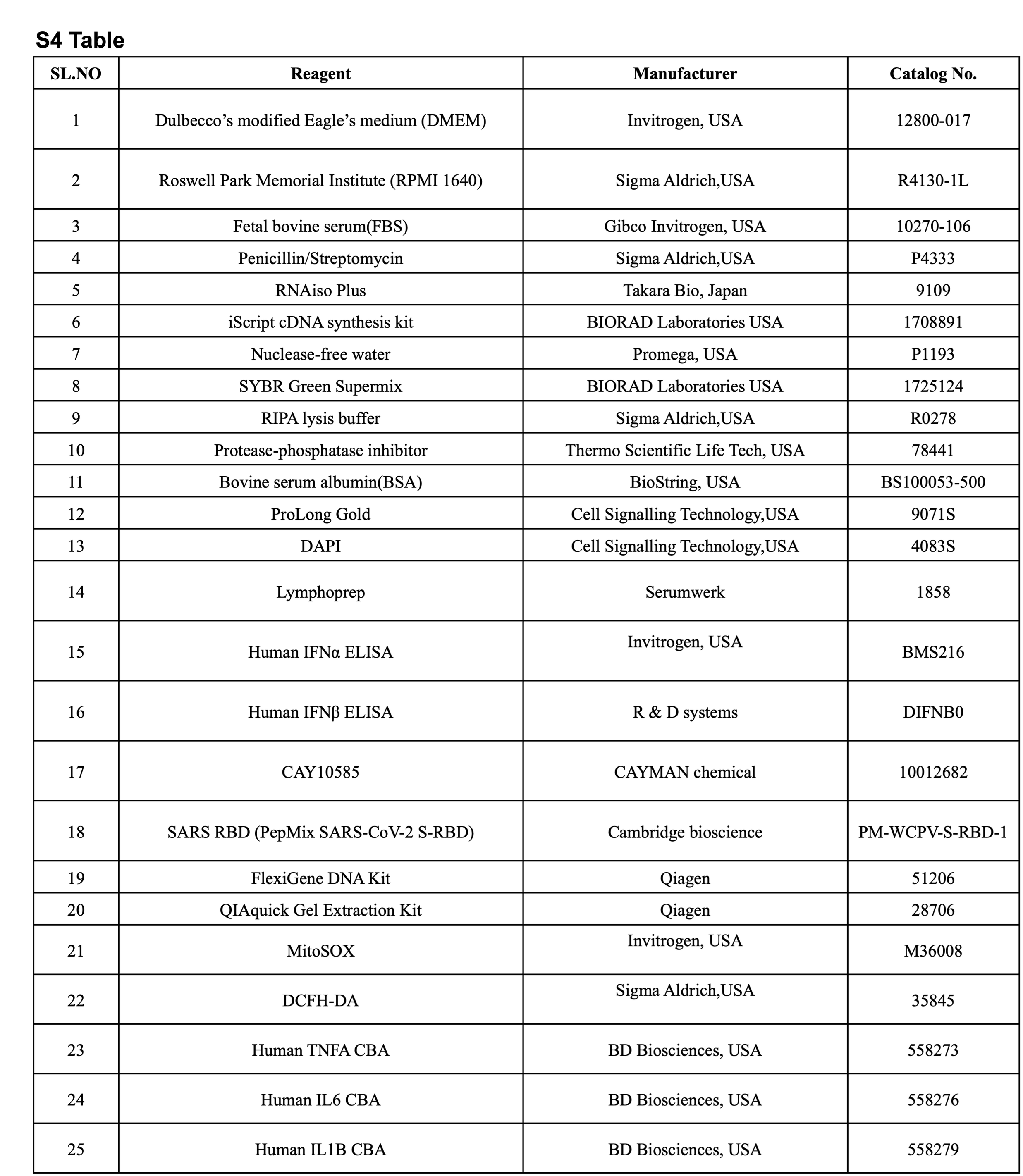

Supplement: S4 Table — (TIF) [file ppat.1013296.s011.tif]

**Fig 2A**

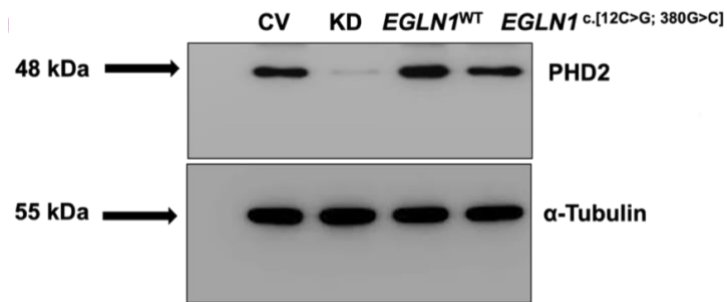

**Fig 2M**

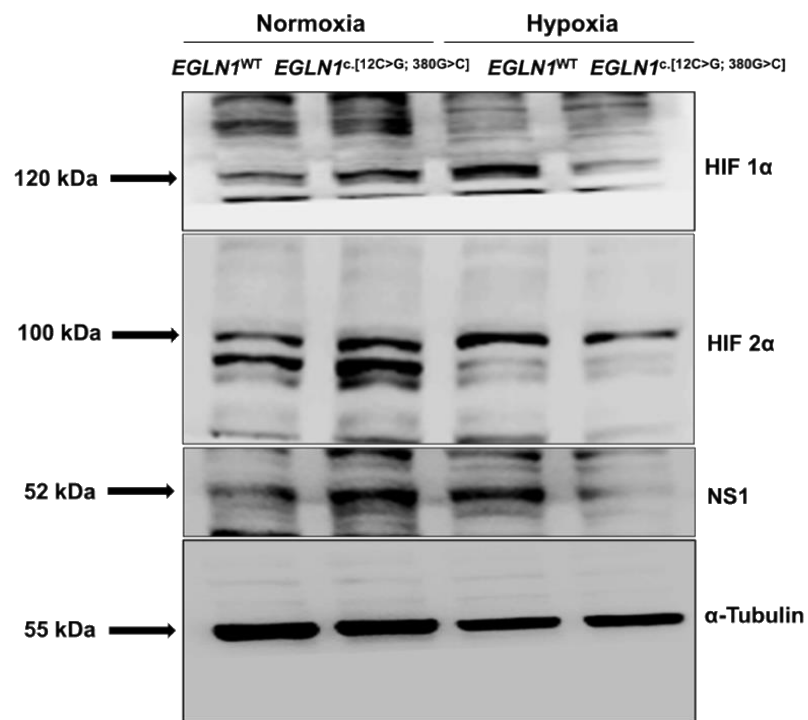

**Fig 3C**

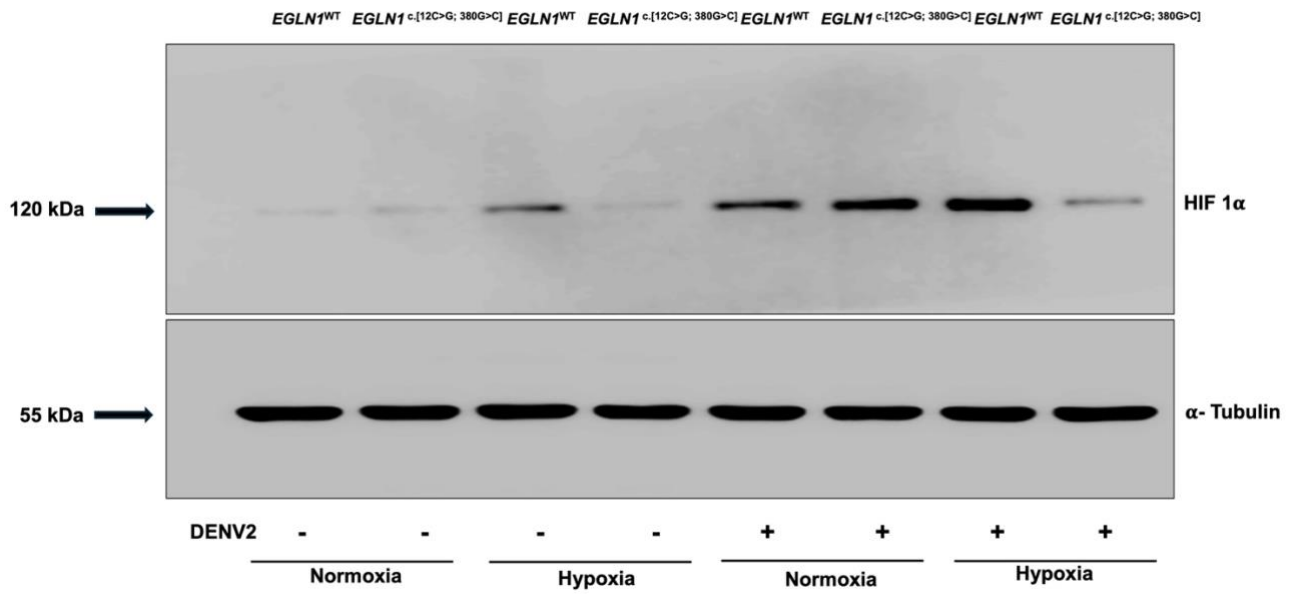

**Fig 3F**

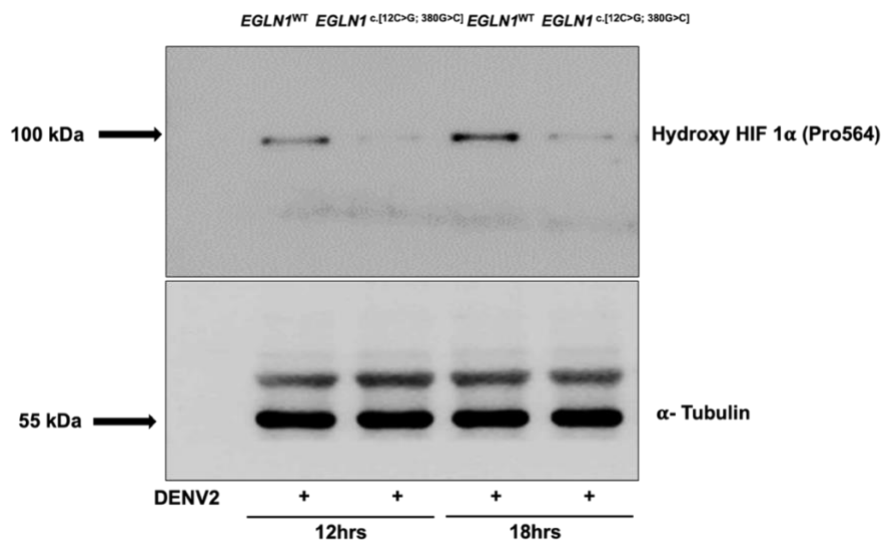

**Fig 4B**

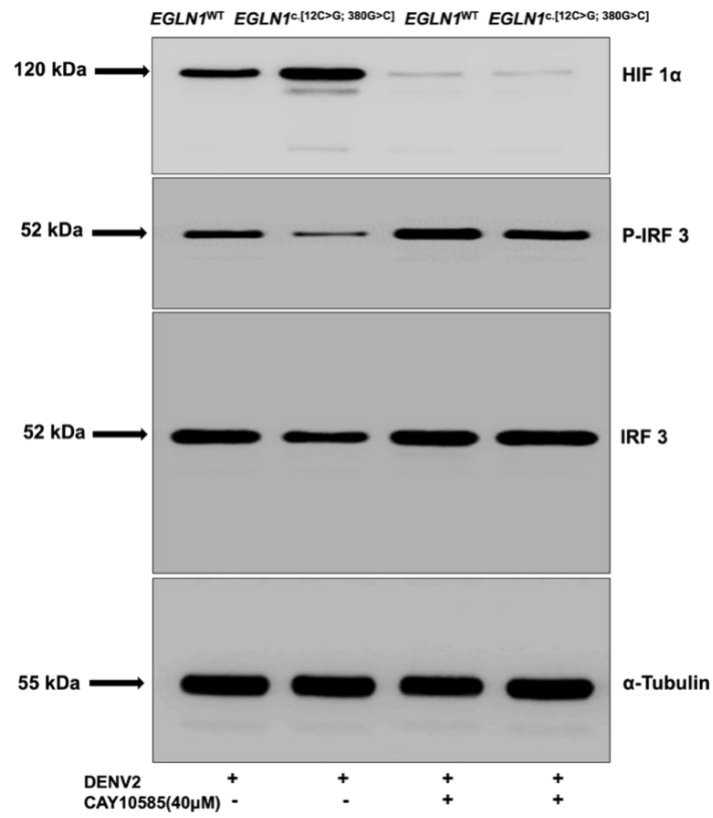

**Fig 5A**

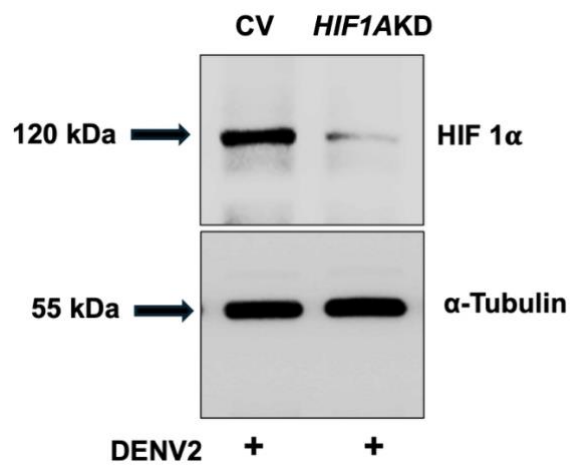

**S3A Fig**

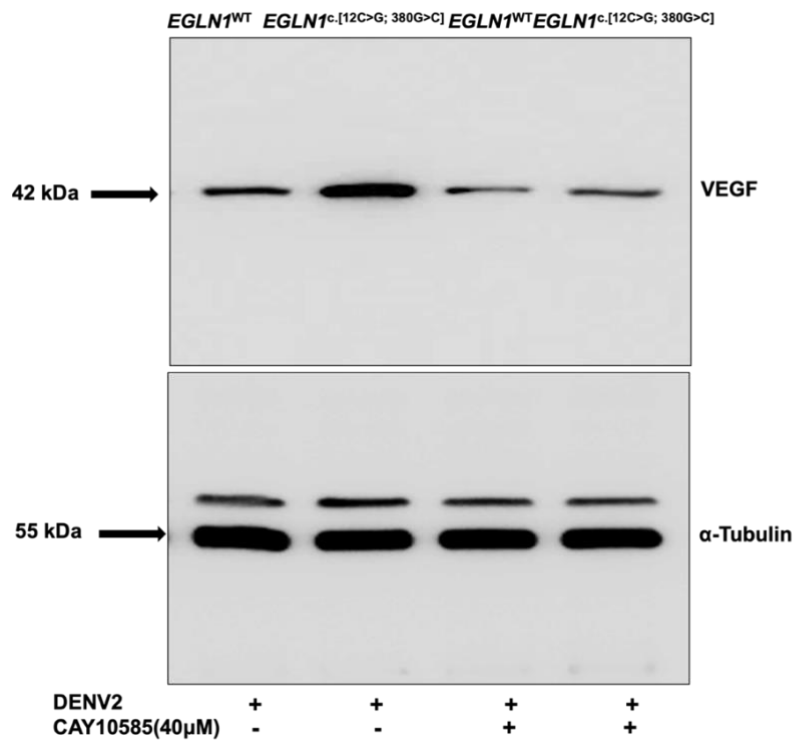

Supplement: S3 File — (PDF) [file ppat.1013296.s014.pdf]
