# Supplementary material for: Tibetan PHD2D4E;C127S variant protects from viral diseases in hypoxia, but predispose to infections in normoxia via HIFα:IFN axis
Source: PLoS Pathog. 2025 Jun 26;21(6):e1013296. doi: 10.1371/journal.ppat.1013296 (PMC12212868; doi:10.1371/journal.ppat.1013296)
Supplement: Attachment — Submitted filename: Reply to reviewer_Revision_F.pdf [file ppat.1013296.s016.pdf]

8PLOS Pathogens: Decision on your submission PPATHOGENS-D-24-02260 - [EMID:ae9aab713ea5a68f]

Reply to Reviewers' Comments:

Part I - Summary
Please use this section to discuss strengths/weaknesses of study, novelty/significance, general execution and scholarship.

Reviewer #1: The authors characterise the impact of a mutant form of PHD2 with a high prevalence in Tibetans previously described to possess a higher affinity for O2 compared to the Wt form resulting in enhanced degradation of HIF-alpha subunits even in reduced oxygen conditions.

Given the above when examining HIF protein expression by immunoblot in normoxic conditions elevated levels of HIF-1 are observed (Figs 2K,3B,5D,J) in cells possessing the mutant form of PHD2 as well as in control wells. Did the authors perform viability assays to confirm the health of the cells in all experiments as HIF expression in otherwise normoxic conditions is symptomatic of poor cell culture health and/or over confluency. Further the increased expression of HIF in PHD2-mutant cells seems incongruent with its reported increased hydroxylation activity, as one would expect decreased expression of HIF not elevated levels at baseline. Could the authors validate whether levels of hydroxylated HIF are indeed augmented by the mutant form of PHD2?

Reply: We appreciate your comments. We measured hydroxylated HIF1α (Pro564) using an antibody (D43B5, Cell Signalling Technology, USA). As mentioned in Fig. 3F-G, the level of hydroxylated HIF1α was lesser in $PHD2^{D4E;C127S}$ than $PHD2^{WT}$ cells at 12 or 18 hrs after DENV2 infection under Normoxia.
As pointed out by the reviewer, we have checked almost no expression of HIF1α in these cells under normoxia without viral infection. However, the HIF1α expression was increased following viral infection (Fig 3C).

Further I have concerns over the approach taken by the authors to overexpress the Tibetan variant of PHD2 (as described in Sfig2). They appear to have transiently knocked down the endogenous form of PHD2 by siRNA followed by overexpression of the variant form. Their siRNA knockdown show a partial reduction in endogenous PHD2 which raises concerns that their subsequent overexpression would resulting in a mixed population of both Wt and mutant PHD2 in the same cell population. The authors should either confirm the relative levels of either form or better yet use CRISPR to generate PHD2 KO cells as is widely reported and then over express either Wt or Mutant PHD2. This would provide a much ‘cleaner’ system to study the specific impact of this mutant form of PHD2. The data presented using the system is in my opinion confounded by their approach raising concerns over the validity of their findings with their exisiting overexpression models. Whilst the PBMC data presented in figure 1 is compelling these findings need to be validated in a more scientifically robust genetic model.

Reply: We agree with the above suggestion of using other methods of making PHD2-KO cells. However, we used shRNA to deplete endogenous PHD2 in these cells, before knocking in the

mutant and WT PHD2 constructs. This is a standard protocol in our lab, adopted from Josef T Prchal's lab (co-author), which was used in our previous publications (Lorenzo FR et al. 2014, reference no. 11; Bhattacharya S et al. 2021, reference no. 20; and Shrimali NM et al. 2021, reference no.23). We have used the new replicative western blot image for depletion of endogenous PHD2 in Supplementary Figure (S2C Fig).
We have validated the insertion of mutant/WT PHD2 in these endogenous-PHD2-depleted cells, using gene sequencing (S6A-S6D Fig).
The primary PBMC data, collected from the homozygous/WT individuals, reflects the similar observation like of the engineered mutant/WT PHD2 cells, showing the influence of PHD2/HIF1α axis on the IFNα synthesis in response to viral infection.

Finally the animal experiment in Sfigure 3, lacks key parameters such as pathology scores and/or immunohistochemistry for viral antigen in lung sections as well as infectious viral titres. The authors also present little data validating the inhibitor used resulted in the expected effect in terms of HIF target gene expression. Whist modulation of innate response genes are observed (Fig5 J-P) no functional validation is provided that the inhibitor is reducing HIF-target gene expression or inhibiting HIF activity. As presented, the phenotypes observed cannot be ascribed to inhibition of HIF activity without further evidence the inhibitor is working as expected.

Reply: We have not included animal experiment in this study.
We have validated the effect of HIF1α-inhibitors, CAY10585 on HIF1α-target proteins like VEGF, in supplementary Figure (S4A and S4B).

Reviewer #2: In the manuscript entitled "Tibetan PHD2D4E;C127S variant protects from viral diseases in hypoxia, but predisposes to infections in normoxia via HIFα:IFN axis" by Ghosh et al., authors have investigated the role of the Tibetan-specific variant of prolyl-hydroxylase-2 (PHD2) D4E;C127S in immune response protection against dengue virus infection. They demonstrated that monocytes expressing PHD2-D4E;C127S promote IRF3-dependent production of IFNα/β under hypoxic conditions by suppressing HIF1α expression. Conversely, under normoxic conditions, these mutant cells fail to suppress HIF1α, leading to decreased synthesis of IRF3/IFNs and increased DENV2 infection. A similar pattern was also observed in the context of SARS-CoV-2 infection. The authors further confirmed the crosstalk between the PHD2D4E;C127S variant and the HIF1α-IFN axis using a HIF1α inhibitor and by expressing the variant in CD4/CD8-T cells. While the findings presented in the manuscript are intriguing, the poor explanation of results and concerns regarding data presentation and the timing of data analysis necessitate major revisions.
Reply: We have performed new experiments and explained the results and revised the discussion parts of the manuscript.

Reviewer #3: The manuscript by Ghosh et al describe experiments that show that the cells expressing the PHD2D4E;C127S variant of prolyl hydroxylase 2 are more susceptible to Dengue or SARS Cov2 infection in normoxia but show increased infection hypoxic conditions. The authors show that the PHD2D4E;C127S variant reduces Hif1 levels in hypoxic conditions and increases the interferon A and B pathway to regulate viral infection. While the results are interesting and of importance and suggest an adaptive advantage to the Tibetans, who carry this variant. The results seem to be a little unclear and incomplete to me as some of the key experiments and details of the quantitative methods are not optimal. It was previously known that the PHD2 variant has higher sensitivity to Oxygen and is capable of degrading Hif1 even

when the pO2 levels are low. In the present manuscript the authors try to link the altered levels of Hif1 to the immune response against Virus in terms of interferon signalling.
Reply: We have included new data and revised the results and discussion parts of the manuscript.

Part II – Major Issues: Key Experiments Required for Acceptance
Please use this section to detail the key new experiments or modifications of existing experiments that should be absolutely required to validate study conclusions.

Generally, there should be no more than 3 such required experiments or major modifications for a "Major Revision" recommendation. If more than 3 experiments are necessary to validate the study conclusions, then you are encouraged to recommend "Reject".

Reviewer #1: A large number of experiments would be required before the MS is suitable for publication - hence the recommendation to reject
Reply: We have included new data in the revised version of the manuscript.

Reviewer #2:

o The authors should clarify the specific time point chosen for DENV2 infection and subsequent analyses. Given the dynamic nature of HIF1α expression under hypoxic conditions, it is crucial to assess HIF1α levels both before and after viral infection to accurately evaluate its role in the infection process.
Reply: We have checked HIF1α levels both before and after viral infection and included new data (Fig 3C-3D) in the revised version of the manuscript.

o In Figures 1K and 1L, the methodology used to identify HIF1α-positive cells should be detailed. Including a representative image of HIF1α staining, similar to Figure 1B, would provide a clearer understanding of HIF1α expression dynamics during infection.

Reply: We have included the detailed methods of flow cytometry staining for HIF1α in the Methods section.
Also we performed new experiment, and included the microscopy image of the HIF1α positive cells and its MFI in Fig 1M-1N.

o Figures 1K, 1L, and 2K demonstrate increased HIF1α expression in PHD2D4E;C127S variant cells under normoxia, attributed to elevated ROS levels. To further elucidate the mechanism underlying this upregulation, the authors should directly compare ROS levels and HIF1α expression between uninfected and virus-infected monocytic cells. This would help determine the specific source of ROS and its causal role in HIF1α activation.

Reply: We appreciate your comments. We have measured the HIF1α and ROS level in the uninfected and infected cells under normoxia and hypoxia and included as Fig 3A-C. Also we included a positive correlation graph between HIF1a expression and ROS level in the Fig 3E. These experiments were stopped at 24 hrs.
To understand the mechanism further, the expression of hydroxylated HIF1α (Pro564) was measured at 12 and 18 hrs after DENV2 infection under normoxia. Importantly, a negative correlation was observed between hydroxylated HIF1α and ROS levels, Fig. 3F-3I.

o In Figure 4B, treatment with the HIF1α inhibitor (CAY10585) under normoxia enhances p-IRF3 expression in both EGLN1WT and EGLN1c(12C>G; 380G>C) cells, while total IRF3 protein levels remain unchanged. However, Figure 4G shows an increased IRF3 gene expression in EGLN1WT cells after CAY10585 treatment, with a more pronounced effect in EGLN1c(12C>G; 380G>C) cells. The authors should provide an explanation for the apparent discrepancy between gene expression and protein levels.

Reply: Thanks for pointing out the same. Indeed, there is elevated IRF3 (total protein). We have replaced the IRF3 (total protein) from another representative blot, in Fig 4B and also added densitometry data in S3D-3F Fig.

o The gain-of-function EGLN1c(12C>G; 380G>C) variant fails to inhibit HIF1α expression under normoxic conditions with elevated ROS levels. It is important to investigate whether ROS affected variant-mediated HIF1α hydroxylation or its subsequent ubiquitin-mediated degradation.

Reply: Please find our reply to your above comments.

o While the study primarily focuses on HIF1α-mediated inhibition of the IRF-IFN axis, it is possible that other pro-inflammatory cytokines independent of HIF1α may contribute to protection against viral infection in Tibetan individuals. Have authors explored any such direction?
Reply: We have measured pro-inflammatory cytokines (TNFα, IL6 and IL1ß, gene and protein levels) in DENV2 and SARS-CoV-2 infected *EGLN1*^{WT} and *EGLN1*$^{c.[12C>G;\ 380G>C]}$ cells under normoxia and hypoxia. We did not observe significant difference between the infected cells of both phenotypes under normoxia or hypoxia, (S5A-5I Fig.).

Reviewer #3:

1. In Fig3 The authors show an elevated ROS levels in variant compared to wt in normoxia upon viral infection it would be interesting to also assess the ROS levels without viral infection. Do the authors have any suggestions on how ROS levels are regulated differentially in these two variants

Reply: In connection to our reply to a similar query of the reviewer-1, we described that the elevated HIF1α expression is correlated with a decreased hydroxylated HIF1α in mutant cells after viral infections under normoxia. The elevated level of ROS inhibited the function PHD2 in hydroxylation of HIF1α in the mutant cells upon infection under normoxia, (Fig. 3A-I).

2. In Fig5 the KD of hif1 seems to have a very modest effect on the viral RNA levels in hypoxic conditions while the interferon gene stimulation seems to be as much as seen in other experiments. In addition, it would have been better to also show the effect of Hif1KD in normoxic conditions too.
In Fig6 the experiments are performed only in normoxic conditions It would be better to show a contrast between normoxic and hypoxic conditions in this case too.
Reply: We added new data in the Fig 5A. The repeated experiments confirmed the reduced viral replication in these HIF1α -KD cells (Fig 5A, ***p<0.001).

We have included western blot data of Hif1KD under normoxic condition in (S2B Fig). All experiments in the Fig 6 describe the SARS-CoV-2 experiment under normoxic condition, was performed under BSL3 facility. Our BSL3 facility does not have the hypoxia chamber. Therefore, SARS-CoV-2 experiments under hypoxic condition is a limitation for us.

3. Very interesting data in Fig7. As it shows that IfnG positive CD4 and CD8 increases in response to peptide challenge. The significance of the differences observed in cells without peptide should also be indicated as it seems that even without challenge of the peptide, the levels of the IfnG cells alters between the Wt and the variant. It would be interesting to see if these differences are also observed in individuals not exposed to the virus or vaccinated, although it would be a challenge to find such samples.
Reply: At this moment, It's a limitation for this study. We do not left with sufficient PBMCs from mutant/WT Tibetan individuals to repeat this experiment without the peptide challenge. Another limitation of this study at this time; we do not have any PBMCs from individuals without vaccination or SARS-CoV-2 infection. In fact, this would been a good observation for this study.

Part III – Minor Issues: Editorial and Data Presentation Modifications
Please use this section for editorial suggestions as well as relatively minor modifications of existing data that would enhance clarity.

Reviewer #1: Not applicable
Reviewer #2: o In the result section "An inhibitors to HIF1α rescued", please correct the sentence.
Reply: We have corrected it.

Reviewer #3: 1. In figures expressing the virus RNA levels that Y axis mentions that as compared to the mock (I am assuming infection). I am not sure that is the correct way to express as a better way would be to express them as a ration of DENV RNA to housekeeping gene, as the mock infected cells should not be having any DENV RNA.
Reply: We have changed the Y axis in Fig 1A, Fig 2A, Fig 4A, Fig 5A, Fig 6A, Fig 6K.

2. Can the authors explain why the DENV RNA levels as per the graph is increased by 10 fold (1 log 10 unit) between PHD2 wt and the variant in normoxic conditions (Fig1A and Fig2A), while the MFI of the viral protein stain show only a modest increase of 50-100% (Fig1C and 2C)
Reply: We have replaced both the microscopy images (Fig 1B and Fig 2B) with representative images, and accordingly calculated MFI (Fig 1C-2C).

3. Was the U937 and HuH7 cells sequenced to verify the alleles that are expressed in these two cell lines that are being used if not they need to be verified
Reply: We have sequenced both mutant cell lines and confirmed the insertion of mutations (S6A-6D Fig.)

4. For Hif1 inhibition the inhibitor conc. Used is 40 microM while a lower concentration is (10 microM) is typically used for inhibiting Hif1a. any reasons for using such high concentrations. Further it is not clear whether the cells were in hypoxic or normoxic conditions in these experiments, Ideally it should be performed in both conditions.

Reply: We have standardized the conc. of CAY10585 in U937 and Huh7 cell line and performed all experiments at 40µM conc. Several publications also mentioned the usage of this drug in vitro, concentration ranging between10µM-90 µM; Leiherer A et al 2014, doi.org/10.1016/j.mce.2013.11.009; Basu RK et al 2011, doi:10.1152/ajprenal.00335.2010; Kang Y et al 2020, doi:10.1016/j.jid.2020.01.036

Figure resubmission:

While revising your submission, please upload your figure files to the Preflight Analysis and Conversion Engine (PACE) digital diagnostic tool, https://pacev2.apexcovantage.com/. PACE helps ensure that figures meet PLOS requirements. To use PACE, you must first register as a user. Registration is free. Then, login and navigate to the UPLOAD tab, where you will find detailed instructions on how to use the tool. If you encounter any issues or have any questions when using PACE, please email PLOS at figures@plos.org. Please note that Supporting Information files do not need this step. If there are other versions of figure files still present in your submission file inventory at resubmission, please replace them with the PACE-processed versions.

Reproducibility:

To enhance the reproducibility of your results, we recommend that authors of applicable studies deposit laboratory protocols in protocols.io, where a protocol can be assigned its own identifier (DOI) such that it can be cited independently in the future. Additionally, PLOS ONE offers an option to publish peer-reviewed clinical study protocols. Read more information on sharing protocols at https://plos.org/protocols?utm_medium=editorial-email&utm_source=authorletters&utm_campaign=protocols
