# Supplementary material for: Tibetan PHD2D4E;C127S variant protects from viral diseases in hypoxia, but predispose to infections in normoxia via HIFα:IFN axis
Source: PLoS Pathog. 2025 Jun 26;21(6):e1013296. doi: 10.1371/journal.ppat.1013296 (PMC12212868; doi:10.1371/journal.ppat.1013296)
Supplement: Attachment — Submitted filename: Reply to reviewers_R2.pdf [file ppat.1013296.s017.pdf]

PPATHOGENS-D-24-02260R1
Tibetan PHD2D4E;C127S variant protects from viral diseases in hypoxia, but predispose to infections in normoxia via HIFα:IFN axis
PLOS Pathogens
……………………………………

Reviewers' Comments:

Reviewer's Responses to Questions

Part I - Summary
Please use this section to discuss strengths/weaknesses of study, novelty/significance, general execution and scholarship.

Reviewer #2: (No Response)

Reviewer #4: The researchers examine the influence of a PHD2 variant (D4E;C127), previously recognized as an adaptive mutation in Tibetans living at high altitudes with restricted oxygen levels, on the cellular susceptibility to infections by dengue virus (DENV) or SARS-CoV-2. This variant demonstrates a stronger affinity for oxygen than the original protein and sustains its enzymatic activity under various low-oxygen conditions, which results in the degradation of the HIF-alpha subunit. Whilst the premise is intriguing there is too much ambiguity in the written text and concerns with the presented data to warrant publication without major revisions

---

Part II – Major Issues: Key Experiments Required for Acceptance
Please use this section to detail the key new experiments or modifications of existing experiments that should be absolutely required to validate study conclusions.

Generally, there should be no more than 3 such required experiments or major modifications for a "Major Revision" recommendation. If more than 3 experiments are necessary to validate the study conclusions, then you are encouraged to recommend "Reject".

Reviewer #2: (No Response)

Reviewer #4: The PHD2 mutant (D4E;C127) has been previously reported to retain a higher affinity for oxygen and thus retain its hydroxylation activity in lower oxygen concentrations compared to WT, thus reducing HIF-1 stabilisation. Whilst this is observed in some experiments (fig3C), in nomoxia expression of the mutant PHD appears to result in HIF-1 stabilisation, which is more pronounced with DENV infection. The approach taken to express this mutant PHD is convoluted, shRNA depletion followed by over expression. What is lacking in all experiments is confirmation of the level of expression of the Wt or Mutant PHD2. This should be included as currently it is not clear whether the changes in HIF-1 expression are a result of incomplete depletion of the endogenous PHD2 or overexpression?

Reply: We have used shRNA to deplete endogenous PHD2 in these cells, before knocking in the mutant and WT PHD2 constructs. This is a standard protocol in our lab, adopted from Josef T Prchal's lab (co-author), which was used in our previous publications (Lorenzo FR et al. 2014, reference no. 11; Bhattacharya S et al. 2021, reference no. 20; and Shrimali NM et al. 2021, reference no.23), and more recently in Shrimali NM & Ghosh R et al. 2025, (DOI: 10.1055/a-2580-0107).

We have replaced the image with another representative image (Fig 3C). We do not find difference in HIF-1 expression between $PHD2^{D4E;C127S}$ and $PHD2^{WT}$ under normoxia without infection.

We have validated the insertion of mutant/WT PHD2 in these endogenous-PHD2-depleted cells, using gene sequencing (S5A-S5B Fig).

For better clarity, we have included the PHD2 engineering protocol and representative PHD2 mutant and WT western blot images in Fig-2A-2B.

Further expression of this mutated PHD2 should increase hydroxylation of HIF not reduce it as seen in fig 3F – can the authors explain this departure with the literature?

Reply: In general mutated PHD2 increases the hydroxylation of HIF in normoxia. However, in Fig 3F, we described that mutant $PHD2^{D4E;C127S}$ failed to promote the hydroxylation of HIF upon DENV infection in normoxia. In mechanism, we described that upon infection, these mutant cells produced more ROS in parallel to mitochondrial dysfunction. More detail of the mechanism is yet to be investigated and we are on it in another work. However, the elevated ROS inhibited the mutant $PHD2^{D4E;C127S}$, in turn a reduced hydroxylation of HIF. The stabilization of HIF correlated with less interferon synthesis in these mutant $PHD2^{D4E;C127S}$ cells upon infection in normoxia as compared with $PHD2^{WT}$ cells. We explained it in the abstract and discussion.

The data presented with DENV is contradictory. HIF is stabilised by DENV as reported in previous work, hypoxic culture increases replication and overexpression of the mutated PHD2 with increased hydroxylation capacity diminishes HIF and thus replication (figure 1). However this does not explain the results observed in normoxia where overexpression of the mutant PHD2 increases DENV replication.

Reply: In hypoxia, both HIF expression and DENV replication increase in WT cells. Others have described a parallel correlation between HIF expression and DENV replication in normoxia as well. Somehow, these mutant $PHD2^{D4E;C127S}$ cells behave differently. As mentioned above, upon DENV infection these mutant $PHD2^{D4E;C127S}$ cells increased ROS generation and inhibited PHD2 function, in turn increased HIF stabilization, and decreased interferon synthesis in normoxia. Thus suppressed interferon in these mutant cells helps DENV replication under normoxia, as compared to WT cells.

These figures lack HIF-1 or PHD1 expression data making it extremely hard to evaluate the validity of these results. Again it is possible that the depletion/over-

expression approach employed here is resulting in contradictory findings. Importantly this is not discussed or mentioned in the text. To validate their findings a model is required with full genetic depletion of PHD2 via CRIPSR should be employed or alternatively CRISPR editing of the endogenous PHD2 to introduce the mutation.

Reply: As mentioned above, we successfully used this protocol of shRNA-mediated depletion of endogenous PHD2 and knocking in the mutant/WT PHD2 in our several recent publications.

For better clarity, we have included the PHD2 engineering protocol and representative PHD2 mutant and WT western blot images of in the Fig-2A-2B.

Reviewer's suggestion is well taken, we are currently developing $PHD2^{D4E;C127S}$ cells using CRISPR technology. It will take some time. We may apply these cells in our future works.

The HIF staining pattern in Figure 1M is irregular, HIF-1 when stabilised should localise to the nucleus not the cytoplasm/perinuclear region.

Reply: We have replaced the Fig 1M with representative images. Due to close timeline, we could not perform new experiments. It's a supporting data of Fig 1L. Data from both flow cytometry and microscopy are showing the similar pattern of HIF expression.

For the HIF silencing experiments presented in figure 5- the level of HIF silencing in these specific experiments should be shown, not just a reference to the exemplar in figure S2.

Reply: We have included the HIF silencing protocol and representative western blot image in the Fig. 5A-5B.

I'm not convinced the SARS-CoV-2 infection data in figure 6 is helpful as the use of the Huh7 cell line is not optimal for this virus. At minimum a respiratory cell line should be employed. Have the authors considered removing this figure and just presenting figure 7?

Reply: As suggested, we have removed Fig 6, and accordingly arranged the results and text.

Part III – Minor Issues: Editorial and Data Presentation Modifications
Please use this section for editorial suggestions as well as relatively minor modifications of existing data that would enhance clarity.

Reviewer #2: (No Response)

Reviewer #4: For all figures the data points should be shown

Reply: We have included the data points in all figures.
